# Redox-Responsive Mesoporous Silica Nanoparticles for Cancer Treatment: Recent Updates

**DOI:** 10.3390/nano11092222

**Published:** 2021-08-28

**Authors:** Miguel Gisbert-Garzarán, María Vallet-Regí

**Affiliations:** 1 Institut Galien Paris-Saclay, UMR 8612, CNRS, Faculté de Pharmacie, Université Paris-Saclay, CEDEX, F-92296 Châtenay-Malabry, France; 2 Departamento de Química en Ciencias Farmacéuticas, Universidad Complutense de Madrid, Instituto de Investigación Sanitaria Hospital 12 de Octubre i + 12, Plaza Ramón y Cajal s/n, 28040 Madrid, Spain; 3 Networking Research Center on Bioengineering, Biomaterials and Nanomedicine (CIBER-BBN), 28029 Madrid, Spain

**Keywords:** mesoporous silica nanoparticles, redox-responsive, stimuli-responsive, drug delivery, cancer cell targeting, biodegradable nanoparticles, periodic mesoporous organosilica nanoparticles

## Abstract

Mesoporous silica nanoparticles have been widely applied as carriers for cancer treatment. Among the different types of stimuli-responsive drug delivery systems, those sensitive to redox stimuli have attracted much attention. Their relevance arises from the high concentration of reductive species that are found within the cells, compared to bloodstream, which leads to the drug release taking place only inside cells. This review is intended to provide a comprehensive overview of the most recent trends in the design of redox-responsive mesoporous silica nanoparticles. First, a general description of the biological rationale of this stimulus is presented. Then, the different types of gatekeepers that are able to open the pore entrances only upon application of reductive conditions will be introduced. In this sense, we will distinguish among those targeted and those non-targeted toward cancer cells. Finally, a new family of bridged silica nanoparticles able to degrade their structure upon application of this type of stimulus will be presented.

## 1. Introduction

Cancer is a leading cause of death worldwide, with 10 million deaths and almost 20 million new cases just in 2020 [1]. Among the different treatment options, chemotherapy involves the administration of cytotoxic compounds able to eliminate the tumoral cells. However, these compounds lack great tumor tissue selectivity, which leads to nonspecific systemic distribution and entails severe side effects.

The application of nanotechnology to medicine, so-called nanomedicine, has undergone an unstoppable development in recent decades. Indeed, nanotechnology has played a major role in the development of vaccines within the current COVID-19 pandemic [2]. Regarding cancer treatment, the use of nanosized carriers seems appealing owing to their capacity to encapsulate drugs within their structure. The rationale behind the use of nanoparticles (NPs) relies on the enhanced permeability and retention (EPR) effect, which promotes their passive accumulation in tumors. The EPR effect is consequence of the impaired tumor blood vessels (200–2000 nm endothelial cell-cell gaps) and the poor lymphatic drainage of such tissues. As a result, NPs leak out from the vessels and remain in the tumor for long periods of time [3]. In addition to this passive tumor targeting, NPs can be decorated with specific targeting ligands able to achieve selective recognition of cancer cells [4] and their subcellular organelles [5], thereby releasing high concentration of drugs within such cells. 

Among the different types of NPs, mesoporous silica-based NPs (MSNs) have attracted much attention as drug carriers, especially since Vallet-Regí and coworkers first reported their suitability to load and release therapeutic compounds in a sustained manner [6]. MSNs offer excellent physicochemical properties, namely (i) a network of hollow cavities with tunable structures and narrow pore size distributions (2–20 nm); (ii) a high specific surface areas (ca. 1000 m^2^/g) and high pore volumes (ca. 1 cm^3^/g); (iii) a high density of silanol groups on the surface; (iv) a resistant silica framework that allows for harsh reaction conditions; and (v) hemo and biocompatibility [7]. 

The open porous structure allows the loading of drugs by simple diffusion and, likewise, such compounds can easily diffuse out of the pores. From an experimental point of view, this premature release minimizes the loading capacity of the NPs, which would entail the need for administering higher doses of nanomedicines. From a clinical perspective, such uncontrolled release would result in the presence of high amounts of free drugs in the bloodstream, thereby causing the issues ascribed to traditional chemotherapy. 

Bearing all that in mind, the efforts of many nanotechnologists have been headed toward the development of structures able to seal the pore entrances under physiological conditions and open them in the tumors. In this regard, drug release can be triggered on-demand upon application of a stimulus. Such stimuli can be externally applied (e.g., magnetic fields, ultrasounds, light) or can rely on some relevant biomarkers that may be upregulated/downregulated in tumor tissues (e.g., pH, enzymes, redox species) [8].

Rather than focusing on classical articles on redox-responsive NPs, this review is intended to provide a comprehensive description of the recent updates on redox-responsive MSNs for cancer treatment. The review starts by describing the physiological basis of redox-triggered drug delivery. Then, the use of gatekeepers that are sensitive to overexpressed glutathione (GSH) is presented. Afterwards, the concept of cancer cell targeting is explored as a manner to eliminate specifically cancer cells. In this sense, two scenarios will be presented: small targeting agents attached to redox-responsive gatekeepers and redox-gatekeepers that can also act as targeting agents. Finally, a new type of MSNs based on the use of redox-responsive biodegradable precursors will be presented as a way to accelerate their elimination from the organism. 

## 2. Rationale of Redox-Responsive Drug Delivery

**Figure 1:**
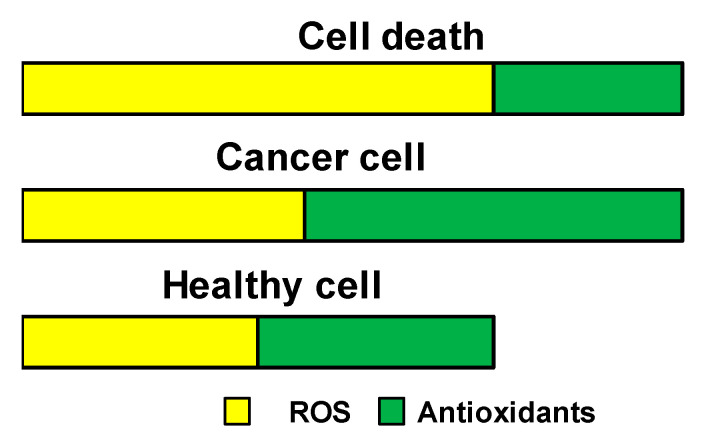
Schematic representation of ROS and antioxidants content in healthy and tumoral cells. The excess of ROS production observed in tumoral cells is counteracted by the production of more antioxidants. However, if the balance is lost, excessive ROS within cells will lead to cell death.

The production of reactive oxygen species (ROS) by aerobic cells is essential, as they mediate crucial intracellular signaling pathways and are vital for cell survival. However, excessive production of ROS, as it happens under conditions of cell injury, leads to cell damage and death. To overcome this, cells produce ROS scavengers (antioxidants), being the major contributor the tripeptide glutathione (γ-glutamyl-cysteinyl-glycine) [9]. The content of ROS and antioxidants in healthy and tumoral cells is schematically represented in Figure 1. 

The most representative ROS actors are superoxide anion radical (O_2_^−^), hydrogen peroxide (H_2_O_2_), and/or hydroxyl radical (·OH). Superoxide anion is enzymatically converted into hydrogen peroxide very rapidly, which shows 10^3^ times longer half-life and can modify the structure and function of proteins through uncontrolled oxidation of their cysteine thiols. In turn, hydrogen peroxide can react with iron ions and produce short-lived hydroxyl radicals through a Fenton reaction. The latter present high toxicity and can induce irreversible damage to nucleic acids, lipids, and proteins. Of note, most tumoral cells have a higher ROS set point than that of associated healthy cells, which may contribute to tumor growth, proliferation, and metastasis [10,11]. 

As mentioned above, GSH plays a vital role in the maintenance of appropriate levels of ROS within cells. It is the most important non-enzymatic antioxidant in cells and GSH/GSSH is the major redox couple in animal cells. The levels of GSH vary throughout the organism. While the levels of GSH in the blood and extracellular matrices are low (2–20 µM), the presence of high levels of GSH (0.5–10 mM), especially in the mitochondria and cytosol, is a general feature of cells [12]. Even though GSH is the major contributor, the redox-active nature of the lysosomes has also been reported. This reductive behavior is controlled by the gamma-interferon-inducible lysosomal thiol reductase, which is especially active at the acid pH of the lysosomes [13]. 

Though it is true that tumor tissues present higher concentrations of GSH compared to normal tissues [14], the levels still seem to be in the µM range and would therefore not constitute a differential feature that could be exploited to deliver drugs specifically at the extracellular tumoral matrix. 

## 3. Redox-Responsive Gatekeepers

As mentioned in Section 2, the presence of high levels of GSH is an overall characteristic of cells. That physiological feature can be exploited for the design of redox-responsive MSNs. These NPs are commonly based on the use of bonds that can be cleaved in the presence of high concentration of redox species. Examples of such bonds include diselenide (–Se–Se–), disulfide (–S–S–), platin conjugation (–Pt–), thioether (–S–), and thiol group (SH) [15], with the disulfide bonds being the most employed.

Those bonds can be employed to engineer different types of gatekeepers on the surface of MSNs. In this manner, the pore entrances would remain sealed in the bloodstream, where the low GSH levels found there are unable to break the bonds. On the contrary, upon cellular internalization, the high production of GSH in the cytosol would cleave such bonds, opening the pores and triggering the drug release. In consequence, a conveniently engineered redox-responsive system should be able to minimize the presence of free drugs in the bloodstream, thereby avoiding common side effects of conventional chemotherapy.

### 3.1. GSH-Degradable Gatekeepers

The first approach involves the use of gatekeepers that present redox-cleavable bonds throughout their backbone. For instance, keratin is the major structural fibrous protein to form hair. It can be employed to seal the mesopores of MSNs, as it presents cysteine residues throughout its structure able to form disulfide bonds, providing stimuli-responsiveness and non-immunogenicity [16]. A rather simple strategy to avoid premature drug release involves taking advantage of having a silica framework to promote further silica condensation. In this sense, Shen et al. coated small MSNs with a nonporous layer of silica made from 3-mercaptopropyltrimethoxysilane grown directly on the surface, obtaining almost zero release in the absence of GSH [17]. Similarly, dopamine can be modified so as to form dimers connected through disulfide bonds. In this way, such dimers can polymerize around the surface of the NPs, leading to polydopamine-coated MSNs sensitive to both pepsin and GSH [18].

**Figure 2:**
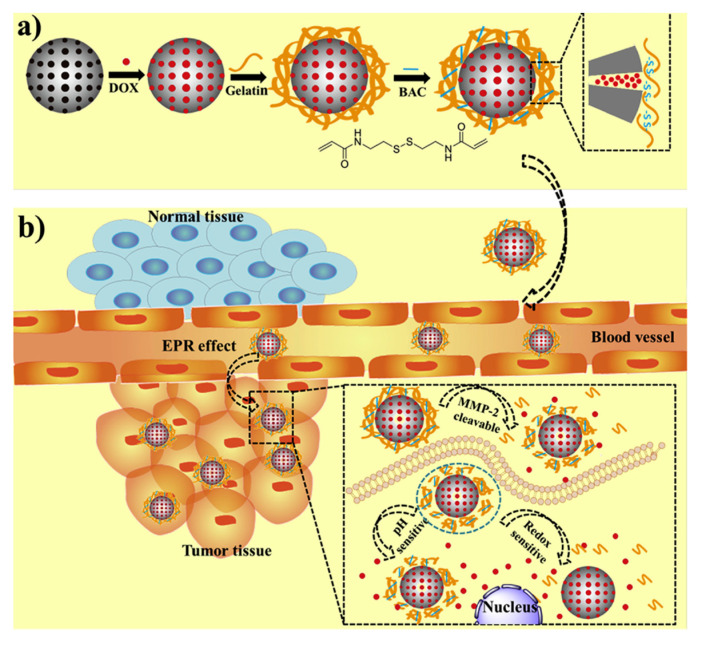
(**a**) Schematic representation of the synthesis of the enzyme- and redox-responsive MSNs. (**b**) Schematic representation of the mechanism of action. The overexpressed metalloproteinases can partially degrade the gelatin coating, which can be completely degraded due to the action of GSH. Reproduced with permission from [20]. Copyright Elsevier, 2019.

Another elegant manner to obtain GSH-degradable gatekeepers is to crosslink in situ a gatekeeper that had previously been attached to the surface. For instance, dextrin can be oxidized to dialdehyde dextrin to form a pH-responsive nanocarrier through Schiff bases. Afterward, crosslinking the carboxylic acid pendant groups of the dextrin derivative with cysteamine dihydrochloride yields a highly compact layer, sensitive to both acid pH and redox species [19]. Enzyme- and redox-responsive MSNs can be produced following a similar strategy (Figure 2). 

As shown in Figure 2, the MSNs were coated with a biocompatible gelatin to prevent drug leakage. However, gelatins act more as a diffusion barrier rather than an actual gatekeeper. For that reason, they incorporated a disulfide-containing methacrylate to crosslink the gelatin, shrinking it and allowing the release only in the presence of metalloproteinases and GSH [20]. 

### 3.2. Redox-Responsive Linkers in Drug Delivery

#### 3.2.1. Polymers and Biomacromolecules

The most extended strategy for designing redox-responsive MSNs involves grafting the different gatekeepers to the surface using redox-responsive linkers. For instance, albumin or myoglobin can be employed to prevent premature drug release by attaching them to the MSNs using diselenide bonds, obtaining a nanocarrier highly responsive to both GSH and H_2_O_2_ [21]. Rather than using bulky proteins, the use of small peptidic molecules as gatekeepers has also been reported. In this regard, Li et al. designed bolt-like gatekeepers by grafting a peptidic molecule bearing tryptophan using disulfide bonds [22]. This amino acid was chosen to take advantage of the hydrophobic interactions of their pendant group, which allowed it to seal the mesopores by interacting with the hydrophobic domains of doxorubicin molecules. 

An alternative strategy involves using a therapeutic agent as gatekeeper. In this sense, Lin et al. produced highly cationic MSNs by attaching a dendronized chitosan derivative via disulfide bonds. In this manner, the redox-responsive NPs were able to prevent premature doxorubicin release as well as to transfect p53 gene, resulting in synergistic therapeutic effect in the presence of GSH [23]. Aiming also at achieving synergistic effect, Zhao et al. coated the MSNs with a thiolated siRNA derivative that was not only able to silence Bcl-2 protein and cause mitochondrial dysfunction, but also reduced doxorubicin leakage from the pores in the absence of GSH [24].

#### 3.2.2. Nanovalves

**Figure 3:**
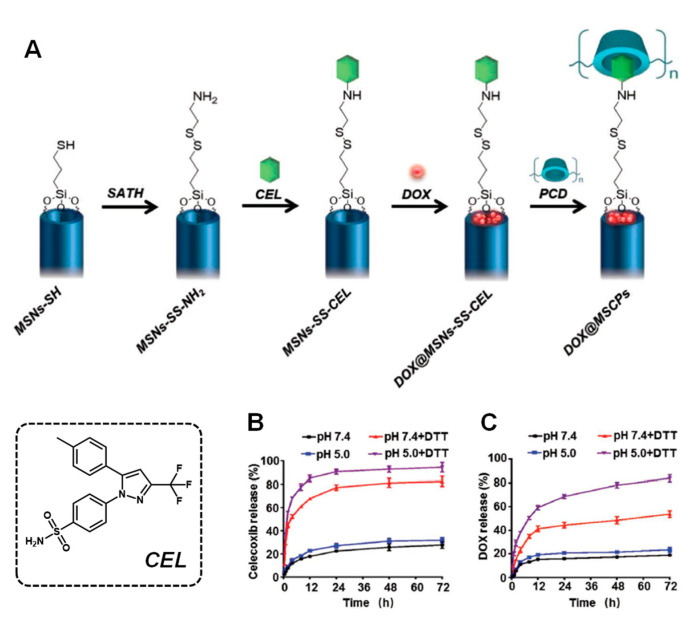
(**A**) Schematic representation of the redox-responsive MSNs. (**B**,**C**) Celecoxib (CEL) and doxorubicin release profiles, respectively, in the presence or absence of dithiothreitol (DTT), which mimics the reductive environment found inside the cells. Adapted with permission from [26]. Copyright Wiley, 2019.

A different approach comprises the use of the so-called nanovalves. These nanovalves are supramolecular structures that tightly close the pores upon interaction with a stalk grafted on the surface. For instance, cucurbit[6]uril (CB [6]) is known to interact with cyclopentyl methylamino through host–guest interactions. Taking advantage of this, Li et al. grafted a cyclopentyl derivative to the NPs, forming a disulfide bond. In this manner, the CB [6] unit was able to seal the pore entrances and further interact with cyclopentyl-modified poly(acrylic) acid to form a redox-responsive, multilayered gatekeeper [25]. Similarly, cyclodextrins (CD) can interact with a range of stalks. For instance, Liu et al. recently reported a smart approach to achieve drug co-delivery and pore gating [26]. The authors designed a stalk for CD based on the drug celecoxib that was attached to the surface via disulfide bonds, avoiding premature release of doxorubicin (Figure 3).

As observed, in Figure 3, the system was able to inhibit premature drug release in the absence of a reductive stimulus, whereas the disulfide bond was cleaved when DTT was added. In this manner, the release of celecoxib blocked the COX-2/PGE_2_ axis, thereby sensitizing the tumoral cells to doxorubicin and abrogating the doxorubicin-induced enhancement of cancer stemness and metastatic capacity.

Of note, not only can the stalk be grafted through disulfide bonds, but it can also be inherently redox-responsive. For instance, Qu et al. modified the MSNs with a small ferrocene-bearing molecule that was able to interact with Au NPs-coated CD [27]. By taking advantage of the high concentrations of H_2_O_2_ secreted by tumoral cells, ferrocene could be oxidized to no longer show affinity for the CD cavity, thereby weakening the interactions and triggering the drug release.

#### 3.2.3. Small Nanoparticles

**Figure 4:**
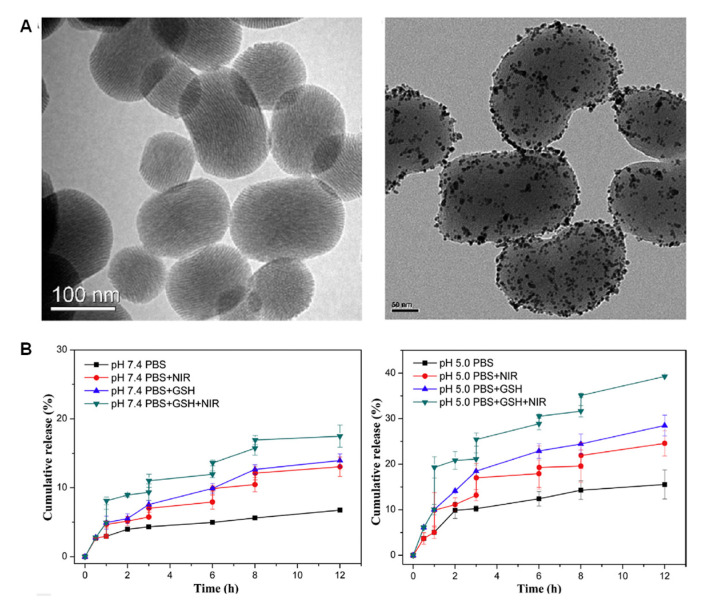
(**A**) TEM micrographs of MSNs and MSNs coated with Au NPs. (**B**) Release profiles at different pHs, in the presence or absence of GSH, and upon application or not of NIR light. The highest drug release is obtained when both GSH and NIR light are applied. Adapted with permission from [30]. Copyright Elsevier, 2017.

The small size of certain types of NPs (<10 nm) makes them ideal candidates to seal the pores entrances of the MSNs. The usefulness of this approach is that such small NPs can exert therapeutic action by themselves. For instance, ZnO quantum dots (QDs) decompose at the pH of the lysosomes, generating cytotoxic Zn^2+^ ions that are known to damage cancer cells. In this sense, Qiu et al. coated the MSNs with such QDs and used oxidized glutathione as a linker to obtain a multi-responsive drug delivery system [28]. Some NPs can generate heat upon application of near-infrared (NIR) light. Following this approach, Gao et al. functionalized the MSNs with graphene QDs through disulfide bonds, demonstrating enhanced drug release when the laser was applied [29]. These two works, yet promising, lack evaluation of their therapeutic effect on cells. In this regard, Yang et al. synthesized Au NPs-capped MSNs and evaluated their performance (Figure 4) [30].

As observed in Figure 4, the gold NPs could be properly grafted through disulfide bonds, which were cleaved in the presence of GSH. Of note, the application of NIR light not only induced cell death through hyperthermia, but also enhanced the amount of drug released, likely due to the hot spot effect [31,32], leading to synergistic therapeutic effect with doxorubicin.

## 4. Small Molecules as Targeting Agents of Redox-Responsive MSNs

Although it has been mentioned that ROS/ROS scavenger levels are higher in cancer cells than in healthy ones, in both cases, the values are within the mM range [11,33]. Hence, not only should MSNs be equipped with smart gatekeepers, but also with recognition ligands that can promote their accumulation specifically in cancer cells. Otherwise, the NPs might accidentally release their content inside the healthy cells, leading to side effects.

Carbohydrates are interesting as targeting agents owing to their ability to interact in a very specific manner with overexpressed lectin proteins present on the cell membrane. In this sense, Wu et al. fabricated light- and redox-responsive MSNs by attaching a UV-sensitive azobenzene through disulfide bonds as stalk. Then, they further modified the CD with galactose to target the asialoglycoprotein receptor, obtaining high efficacy against hepatocellular carcinoma [34].

**Figure 5:**
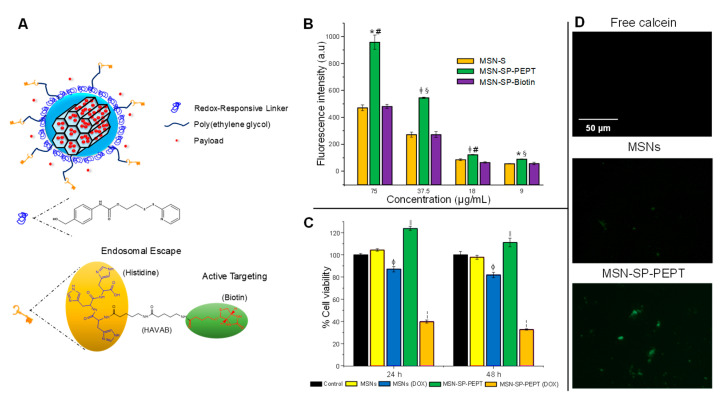
(**A**) Schematic representation of the redox-responsive targeted MSNs. (**B**) Flow cytometry on HeLa cells demonstrated that the highest internalization was obtained for group containing both the biotin and the histidine motifs. (**C**) The redox-responsive, targeted MSNs exerted significant cytotoxicity on A549 cells when loaded with doxorubicin at short time. (**D**) The calcein assay demonstrated that only the group bearing the peptidic molecule was able to escape the endosomes. Adapted with permission from [35]. Copyright ACS, 2021.

Tumors present high requirements of vitamins, which leads to the overexpression of their receptors on tumoral cells. In this sense, our group recently reported targeted redox-responsive MSNs [35]. We incorporated a redox-responsive gatekeeper based on self-immolative chemistry and further functionalize it with a peptidic molecule containing biotin (vitamin B7) for cellular targeting and histidine for inducing endosomal escape (Figure 5).

As observed in Figure 5, the presence of the peptidic molecule enhanced the accumulation in tumoral cells, even observing a synergistic effect between histidine and biotin, leading to great cytotoxicity on cancer cells. In addition, the endo-lysosomal escape was validated with the calcein assay, in which the detection of green fluorescence was indicative of the rupture of such vesicles.

Folic acid (vitamin B9) has long been explored as a targeting agent. For instance, it has been employed to target chitosan-coated MSNs to breast cancer cells, achieving controlled drug release inside the cells [36]. The use of polypeptides as gatekeepers has also been explored. Cui et al. fabricated folic acid-containing thermo-responsive poly(γ-benzyl-L-glutamate) peptides and grafted them to the MSNs through disulfide bonds, obtaining high efficacy against lung cancer cells in the presence of GSH [37]. Following a similar approach, Demirel et al. engineered redox-responsive MSNs by coating them with a poly-L-histidine-PEG-lipoic acid block. Here, the redox-responsiveness was introduced by crosslinking the lipoic acid molecules with DTT to seal the pores. Then, rather than attaching the folic acid to that complex structure, it was PEGylated and grafted along with the responsive block [38]. Aside from polymer-like structures, the pore entrances of folic acid-targeted MSNs can also be closed using some of the structures that have already been described, including CD [39] and Au NPs [40].

## 5. Targeting Agents as Redox-Responsive Gatekeepers

Section 4 provides several examples of redox-responsive MSNs that are endowed with selectivity for cancer cells by attaching small molecules to the different gatekeepers. However, that may introduce much complexity into the synthesis of the final system. For that reason, using targeting agents that can also act as gatekeepers arises as a promising approximation.

### 5.1. Charge Reversal

**Figure 6:**
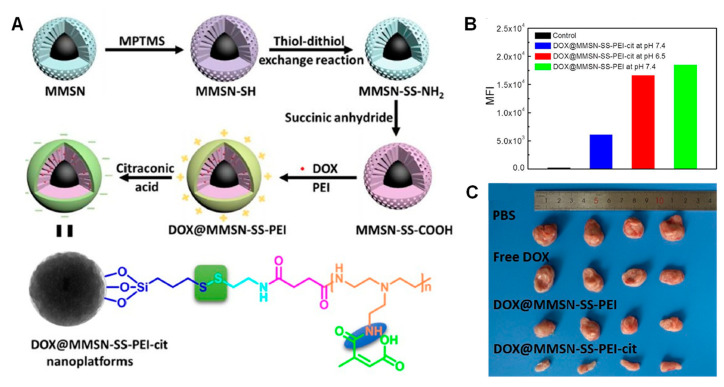
(**A**) Schematic representation of synthesis of the redox-responsive masked MSNs. (**B**) Application of acid pH results in amine exposure and subsequent enhanced uptake. (**C**) The in vivo experiments showed that the system containing the redox- and pH-responsive bonds exerted the highest toxicity. Adapted with permission from [41]. Copyright Elsevier, 2020.

The simplest approximation for triggering the internalization of NPs in cells is having a positively charged surface that is able to interact with the negatively charged cell membrane. However, NPs having positive charge are more prone to undergo opsonization and subsequent bloodstream clearance [8]. A smart approximation is to mask the positive charges of the gatekeeper using pH-responsive bonds cleavable at the pH of the extracellular tumoral matrix (ca. 6.5–6.8). Following this approach, Wan et al. coated the MSNs with highly cationic polyethyleneimine (PEI) through disulfide bonds, and masked the charges using citraconic anhydride [41] (Figure 6).

The PEI coating was able to prevent premature drug release in the absence of GSH and, as observed in Figure 6, the internalization of the masked nanocarriers was much lower than those exposed to acid pH, obtaining high efficacy in vivo. The same authors reported MSNs capped with CD using a stalk attached to the surface through disulfide bonds. Then, they incorporated a polylysine masked with citraconic anhydride, again obtaining redox-responsiveness and enhanced efficacy in vivo after exposing the positive charges at acid pH [42].

### 5.2. Antibodies, Aptamers, Peptides and Proteins

This kind of targeting agent is interesting because their relatively high molecular weight allows them to be used also as gatekeepers. For instance, Chen et al. fabricated gated MSNs by attaching the anti-carbonic anhydrase IX antibody via disulfide linkages [43]. In this manner, they managed to reduce premature drug release in the absence of GSH and achieved great recognition of tumoral cells overexpressing carbonic anhydrase IX, leading to enhanced tumor reduction.

Aptamers show similar targeting ability but they are non-immunogenic and easier to produce. In this sense, Zhuang et al. employed the aptamer AS1411, which targets the nucleolin protein, to engineer a redox-responsive gatekeeper for the MSNs [44]. Rather than merely loading doxorubicin, they incorporated a thiolated siRNA able to inhibit TIE2 to sensitize doxorubicin-resistant breast tumors. Both the aptamer and the siRNA were attached using disulfide bonds, thereby reducing premature drug release and releasing both the drug and the siRNA upon action of GSH in the cytosol.

**Figure 7:**
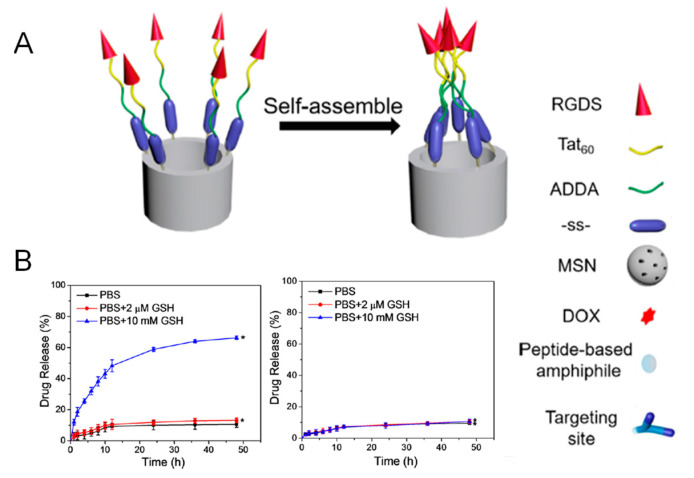
(**A**) Schematic representation of the redox-responsive gatekeeper. (**B**) Release experiment in the presence or absence of biologically relevant levels of GSH. Of note, when a disulfide bond is not employed the drug release does not take place (graph on the right). Reproduced with permission from [45]. Copyright ACS, 2017.

The main advantage of using peptides over other targeting agents is the ease of their production using well-stablished solid-phase peptide synthesis. In this regard, Cheng et al. reported an amphiphilic peptide able to target cancer cells and close the pore entrances at the same time via disulfide bonds (Figure 7) [45].

The peptide was composed by a hydrophobic segment (12-aminododecanoic acid) and a hydrophilic part containing the TAT_48–60_ peptide sequence along with a small RGD peptide. As observed in Figure 7, the hydrophobic interactions between the alkyl chains were responsible for pore sealing. Of note, GSH was only able to trigger the release when the peptide was attached through disulfide bonds, assuring the redox-responsiveness of the system.

Cancer cells have high requirements of iron, which leads to the overexpression of the transferrin receptor to satisfy such demand. Hence, the use of proteins that target such receptor to close the mesopores is highly appealing. For instance, Cai et al. employed recombinant human H chain ferritin to target the Trf1 receptor and grafted it to the MSNs through disulfide bonds [46]. This approach was able to seal tightly the pore entrances in the absence of redox stimulus, while obtaining high targeting and antitumoral efficacy in vitro and in vivo. Similarly, the protein transferrin has also been employed as a dual gatekeeper and targeting agent, and has been successfully used to deliver doxorubicin to Trf1-receptor positive cancer cells in a redox-responsive manner thanks to their attachment via disulfide bonds [47,48].

### 5.3. Carbohydrates

As mentioned above, carbohydrates can be employed as targeting agents for overexpressed lectin proteins. In this sense, rather than using small carbohydrate molecules attached to other gatekeepers, it is possible to employ high molecular weight carbohydrates as both gatekeepers and targeting agents. Indeed, this approach normally leads to enzyme-responsive systems, as these polysaccharides are degraded by enzymes that are relevant in tumors. For instance, Liu et al. fabricated redox-responsive MSNs by attaching chondroitin sulfate through a cysteine to target the CD44 receptor [49].The authors loaded paclitaxel along with quercetin to tackle drug resistance caused by p-glycoprotein. In this manner, the release took place only inside the tumoral cells overexpressing such receptor, where the high concentration of GSH disrupted the disulfide bonds.

**Figure 8:**
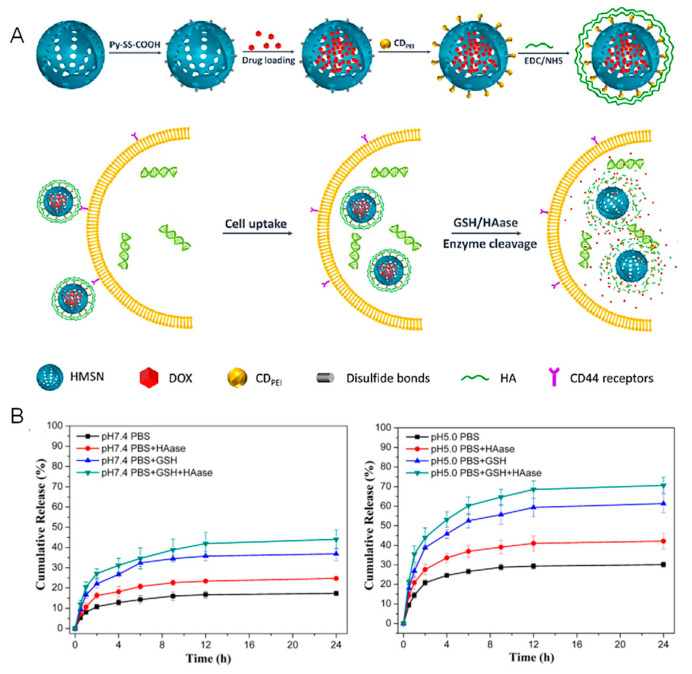
(**A**) Schematic representation of the synthesis of the nanosystem as well as of the mechanism of cellular uptake and triggering of drug release. (**B**) Release experiments in the presence or absence of GSH and hyaluronidase that show that the highest release took place upon application of both stimuli. Reproduced with permission from [53]. Copyright Elsevier, 2017.

Hyaluronic acid has been largely employed as both a gatekeeper and targeting agent for the CD44 receptor. In this regard, there are several methods to incorporate it. For instance, it can be directly attached to the surface through disulfide bonds [50] or it can be incorporated within the structure of a disulfide-containing, cross-linked methacrylate [51]. There are also combinations of carbon dots and hyaluronic acid. For instance, Wang et al. synthesized hyaluronic acid-coated carbon dots, which were subsequently attached to the MSNs using a linker bearing a disulfide bond [52]. Simultaneously, Zhao et al. reported a similar strategy (Figure 8):

As shown in Figure 8, they first attached PEI-coated carbon dots to the surface of the MSNs using a disulfide-containing linker to then incorporate the hyaluronic acid as a layer around the whole surface of the nanoparticles. Then, given that hyaluronic acid can be degraded by hyaluronidase, the presence of that enzyme along with the overexpression of GSH lead to enhanced drug release and targeting toward lung cancer cells [53].

The different types of gatekeepers, as well as whether they are targeted toward tumoral cells, are summarized in Table 1.

## 6. Towards Biodegradability of MSNs: Redox-Responsive Bridged NPs

The US Food and Drug Administration (FDA) considers silica as a “generally recognized as safe” element, and it is often used as dietary supplement and as excipient in drug formulations [54,55]. In this sense, both porous and nonporous silica NPs can be hydrolytically degraded over time into biocompatible, water-soluble silicic acid, which is eventually excreted in the urine [56]. However, the rate of this degradation pathway may vary significantly depending on the size, specific surface area, condensation degree, or surface functionalization, among other factors. This phenomenon can be tuned by introducing bridged organic groups within the framework of the NPs, either alone, along with other bridged organosilane precursors, or in combination with classical silica precursor TEOS [57]. These organosilica precursors consist of an organic functional linker placed between at least two Si atoms. They have a general structure of R[Si(OR’)_3_]_n_ (*n* ≥ 2), being R as the functional linker and R’ as the hydrolyzable group (normally methoxy (–OCH_3_) or ethoxy (–OC_2_H_5_) groups). NPs synthesized by this approach are usually referred to as periodic mesoporous organosilica nanoparticles (PMOS).

The features of those organic groups will determine the dissolution rate of the NPs. For instance, NPs made entirely from benzene-bridged organosilane precursors are extremely stable for at least 6 days (2% weight loss in serum-containing culture medium vs. 80% weight loss for conventional MSNs) [58]. Nonetheless, it would be desirable that bridged NPs degrade in a reasonable time after exerting their therapeutic action. This is an important factor as the effect of intact NPs in the organism for large periods of time could have unpredictable consequences.

Considering that the high concentration of GSH found within cells is able to cleave certain types of bonds, designing bridged nanoparticles containing redox-sensitive bonds stands out as an opportunity to modulate the NPs degradation in living systems. This approach not only results in faster biodegradability rates, but also in accelerated drug release within cells owing to the decomposition of the NPs skeleton. The synthesis of these NPs normally relies on the use of mixtures of biodegradable/non-biodegradable silane precursors (Table 2). Such NPs were produced through the in situ reaction of two silica precursors (3-mercaptopropyl) trimethoxysilane and 3-(thiocyanatopropyl) triethoxysilane) during NPs formation, leading to NPs that showed degradability at 7 days.

**Figure 9:**
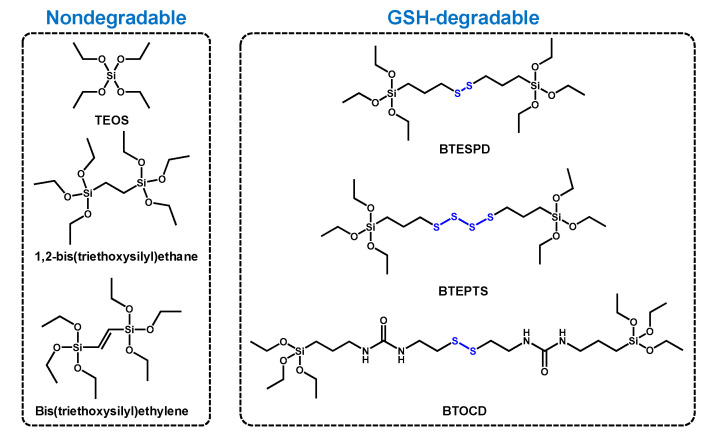
Chemical structure of the different organosilanes employed for the synthesis of biodegradable, bridged mesoporous silica nanoparticles.

The chemical structure of the different organosilanes of Table 1 is illustrated in Figure 9.

The degradation rate seems to be affected by the type of GSH-responsive silane employed. In this sense, Pouya et al. found that the degradation rate of disulfide-containing NPs was faster than that of tetrasulfide-containing ones. A possible explanation might be that the proportion of BTESPD within the framework of the NPs was found to be higher than that of BTEPTS, likely due to the slightly lower molecular weight. Hence, the former had more degradable bonds and, consequently, the dissolution rate was faster [61].

**Figure 10:**
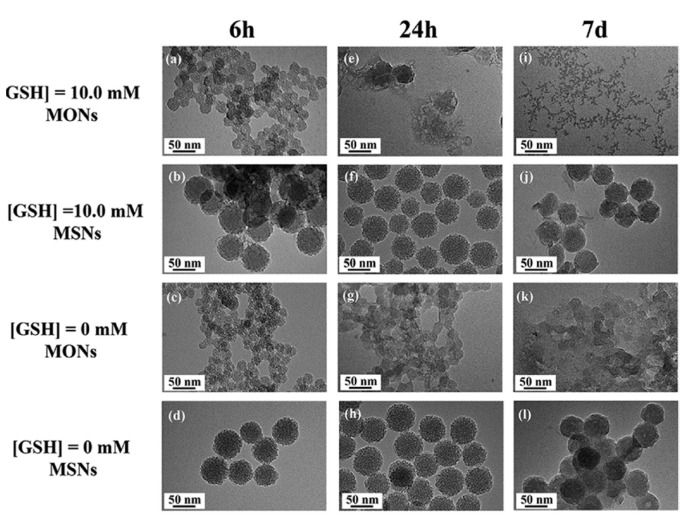
Representative example of the biodegradation by GSH of bridged (here denoted as MONs) vs. conventional MSNs. The different NPs were incubated in SBF solution in the presence or absence of 10 mM of GSH. As observed, conventional MSNs were nearly unaffected after 7 days, irrespective of the presence of GSH, whereas those containing disulfide bonds broke into tiny fragments in the reductive environment. Reproduced with permission from [63]. Copyright Elsevier, 2018.

As expected, the main advantage of this kind of mesoporous silica-based NPs over traditional MSNs is their faster degradation under biologically relevant conditions. A representative example is shown in Figure 10.

As observed in Figure 10, the incubation of the different NPs with SBF in the presence or absence of GSH led to completely different degradation behaviors. Conventional MSNs maintained their overall structure after 7 days. However, disulfide-bridged MSNs depicted first signs of degradation even after 24 h, to further decompose into small fragments after a week. As a general trend, the different articles that have been analyzed for this section show a mean dissolution time of 7–15 days. However, we have also found that many authors tend to focus on the biodegradability of the NPs alone. In our opinion, the focus should be placed on the complete system, as it is known that the surface functionalization delays the degradation of silica-based nanoparticles [70].

Despite being a relatively new field (to the best of our knowledge the first paper on GSH-responsive, bridged silica-based NPs was published in 2014 [71]), there are already available some advanced biological applications of this kind of NPs.

**Figure 11:**
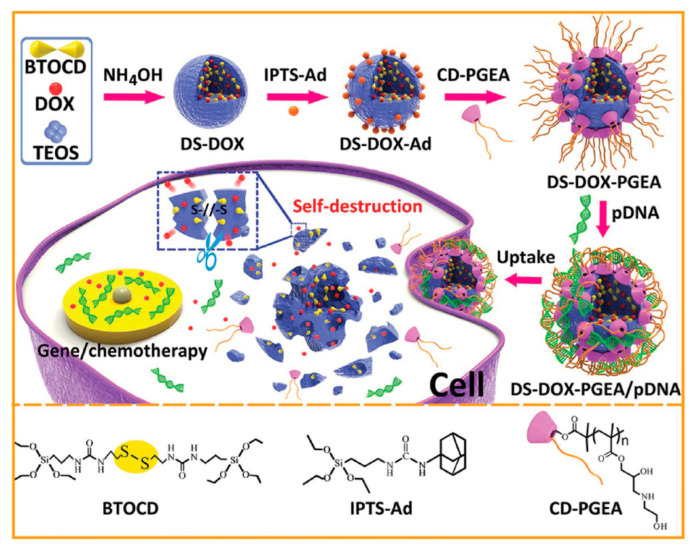
Schematic representation of disulfide-bridged silica NPs for dual doxorubicin and p53 gene delivery. Doxorubicin is loaded in situ during the synthesis of the NPs, which are further modified with a polycation for gene condensation. Upon internalization, the NPs are destroyed by GSH, delivering the drug and the gene. Adapted with permission from [66]. Copyright Wiley, 2017.

For instance, Zhang et al. demonstrated the feasibility of using disulfide-bridged silica nanoparticles for the co-delivery of doxorubicin and plasmid p53, which is able to inhibit tumor cell proliferation (Figure 11) [66].

As shown in Figure 11, these NPs were designed so as to co-deliver doxorubicin and the gene p53. Interestingly, the authors demonstrated that the degradation pattern varied depending on the presence or absence of doxorubicin within the framework. In addition, not only was this system able to inhibit by ca. 80% the viability of C6 cells in just 48 h, but could also target tumors in vivo upon injection in the vein tail, resulting in enhanced tumor growth inhibition.

**Figure 12:**
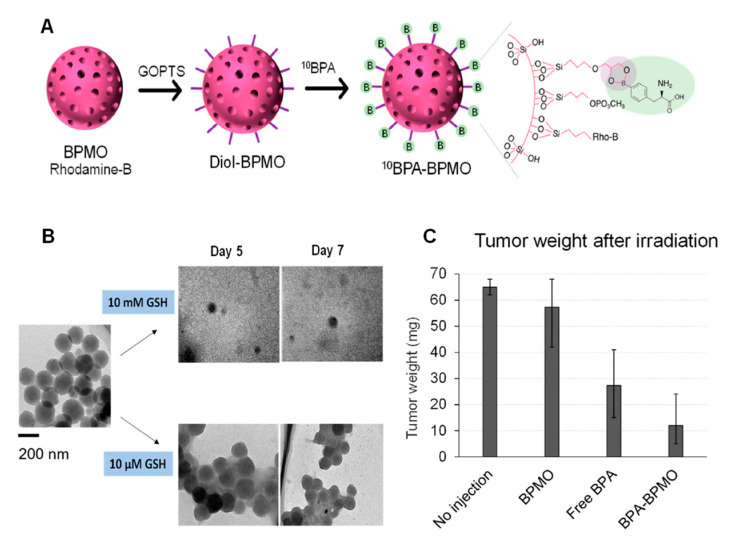
(**A**) Schematic representation of bridged, biodegradable MSNs functionalized with boronophenylalanine on the surface for BNCT. (**B**) The NPs degraded almost completely after 7 days in the presence of GSH. (**C**) Great tumor reduction was observed in the group of irradiated NPs. Adapted with permission from [68]. Copyright MDPI, 2021.

Tamanoi et al. recently reported an innovative approach for the treatment of tumors using biodegradable, bridged mesoporous silica NPs for boron neutron capture therapy (BNCT) (Figure 12) [68]. BNCT involves irradiation of boron-10 with thermal neutrons to produce highly energetic α-particles that cause irreparable injuries in the DNA.

As shown in Figure 12, the authors attached boronophenylalanine to the surface for BNCT. Their experiments showed remarkable tumor reduction after 1 h of irradiation. Of note, the NPs degraded after just 7 days, which would guarantee their fast elimination from the chicken embryos after exerting their action. This approach constitutes a promising application of NPs and should be further explored in the future.

As it has been observed, most of the reported nanosystems involve the use of disulfide (–S–S–) and tetrasulfide (–S–S–S–S–) organosilane precursors to produce thioether-bridged silsesquioxane frameworks. Nonetheless, examples of manganese- or selenide-containing NPs have also been reported. The addition of MnSO_4_ prior to TEOS condensation results in Mn-O bonds that are sensitive to both acid and reductive conditions [72]. Regarding NPs-containing (–Se–Se–) groups, their main advantage over those sulfide-based is that they are sensitive to both reduction and oxidation environments, which should result in faster degradation in cells [73].

## 7. Conclusions and Final Remarks

The use of redox-responsive nanoparticles would guarantee that the drug release takes place within the target cells, as the low concentration of reductive species in the bloodstream would be unable to break the redox-sensitive bonds. Hence, this approach would avoid one of the main drawbacks of chemotherapy, i.e., the presence of free drugs in the bloodstream showing nonspecific systemic distribution. Though there are already some relevant experimental works on this topic, we would like share some concerns that we think should be addressed for this kind of nanoparticles to reach the clinic. First of all, there is the need to scale up the production of this functionalized nanocarriers, which would be the result of the potential collaboration between academia and industry. In addition, the grafting of the different targeting agents should be optimized, as it is known that only a very small percentage of the injected dose of nanoparticles finally reach the tumors. Taking into account these two points, we considered that researchers should aim to design synthetically simple nanocarriers as, otherwise, their industrial production would be compromised, and also their behavior within the body would be rather complex. Finally, even though the redox-responsive, bridged nanoparticles show faster dissolution rates than conventional MSNs, researchers should focus on studying the degradation of coated nanoparticles, which would be much slower. In this sense, we encourage them to produce new bridged structures with accelerated degradation rates that compensate the extra time required for dissolution owing to the presence of the gatekeepers.

## Figures and Tables

**Table 1:** Summary of the different gatekeepers described in the previous sections and whether they are targeted.

Gatekeeper	Targeted [X/✔ (Targeting Agent)]	Target	References
ANTIBODIES, APTAMERS, PEPTIDES AND PROTEINS			
Keratin	X	-	[16]
Albumin; Myoglobin	X	-	[21]
Poly(γ-benzyl-L-glutamate)	✔ (folic acid)	Folic acid receptor	[37]
Poly-L-histidine	✔ (folic acid)	Folic acid receptor	[38]
Anti-carbonic anhydrase IX antibody	✔ (gatekeeper)	Carbonic anhydrase IX	[43]
AS1411 aptamer	✔ (gatekeeper)	Nucleolin protein	[44]
Amphiphilic peptide	✔ (RGD)	αβ-integrins	[45]
Ferritin	✔ (gatekeeper)	TRf1 receptor	[46]
Transferrin	✔ (gatekeeper)	TRf1 receptor	[47,48]
POLYMERS			
Polydopamine	X	-	[18]
Dextrin	X	-	[19]
Gelatin	X	-	[20]
Chitosan	X	-	[23]
	✔ (folic acid)	Folic acid receptor	[36]
Polyethylene imine	✔ (gatekeeper masked with citraconic anhydride)	Exposition of positive charge	[41]
Polylysine	✔ (gatekeeper masked with citraconic anhydride)	Exposition of positive charge	[42]
SMALL NANOPARTICLES			
ZnO quantum dots	X	-	[28]
Graphene quantum dots	X	-	[29]
Au NPs	X	-	[30]
	✔ (folic acid)	Folic acid receptor	[40]
SMALL MOLECULES			
Tryptophan-containing small molecule	X	-	[22]
Self-immolative carbamate	✔ (biotin)	Biotin receptor	[35]
CARBOHYDRATES			
Chondroitin sulfate	✔ (gatekeeper)	CD44 receptor	[49]
Hyaluronic acid	✔ (gatekeeper)	CD44 receptor	[50,51,52,53]
NANOVALVES			
Cucurbit[6]uril	X	-	[25]
Cyclodextrin	X	-	[26]
	X	-	[27]
	✔ (galactose)	Asialoglycoprotein receptor	[34]
	✔ (folic acid)	Folic acid receptor	[39]

**Table 2:** Summary of the biodegradable and non-biodegradable organosilanes employed for the synthesis of bridged NPs.

Non-Biodegradable Silane	Biodegradable Silane	References
TEOS	BTEPTS	[59,60,61,62]
	BTESPD	[61,63,64,65]
	BTOCD	[66]
1-2-bis(triethoxysilyl)ethane	BTEPTS	[67,68]
Bis(triethoxysilyl)ethylene		[69]

TEOS: Tetraethyl ortosilicate; BTEPTS: Bis(3-triethoxysiliyl)propyl) tetrasulfide; BTESPD: Bis(3-triethoxysiliyl)propyl) disulfide. BTOCD: Bridged silane synthesized from 3-(triethoxysilyl)propyl isocyanate and cysteamine dihydrochloride.

## Data Availability

Not applicable.

## References

[1] Sung H., Ferlay J., Siegel R.L., Laversanne M., Soerjomataram I., Jemal A., Bray F. (2021). Global cancer statistics 2020: GLOBOCAN estimates of incidence and mortality worldwide for 36 cancers in 185 countries. CA Cancer J. Clin..

[2] Weiss C., Carriere M., Fusco L., Capua I., Regla-Nava J.A., Pasquali M., Scott J.A., Vitale F., Unal M.A., Mattevi C. (2020). Toward nanotechnology-enabled approaches against the COVID-19 pandemic. ACS Nano.

[3] Maeda H., Nakamura H., Fang J. (2013). The EPR effect for macromolecular drug delivery to solid tumors: Improvement of tumor uptake, lowering of systemic toxicity, and distinct tumor imaging In Vivo. Adv. Drug Deliv. Rev..

[4] Shi J., Kantoff P.W., Wooster R., Farokhzad O.C. (2017). Cancer nanomedicine: Progress, challenges and opportunities. Nat. Rev. Cancer.

[5] Gisbert-Garzarán M., Lozano D., Vallet-Regí M. (2020). Mesoporous silica nanoparticles for targeting subcellular organelles. Int. J. Mol. Sci..

[6] Vallet-Regí M., Rámila A., del Real R., Pérez-Pariente J. (2001). A new property of MCM-41: Drug delivery system. Chem. Mater..

[7] Manzano M., Vallet-Regí M. (2020). Mesoporous silica nanoparticles for drug delivery. Adv. Funct. Mater..

[8] Gisbert-Garzarán M., Vallet-Regí M. (2020). Influence of the surface functionalization on the fate and performance of mesoporous silica nanoparticles. Nanomaterials.

[9] Traverso N., Ricciarelli R., Nitti M., Marengo B., Furfaro A.L., Pronzato M.A., Marinari U.M., Domenicotti C. (2013). Role of glutathione in cancer progression and chemoresistance. Oxid. Med. Cell. Longev..

[10] Kennedy L., Sandhu J.K., Harper M.E., Cuperlovic-culf M. (2020). Role of glutathione in cancer: From mechanisms to therapies. Biomolecules.

[11] Bansal A., Celeste Simon M. (2018). Glutathione metabolism in cancer progression and treatment resistance. J. Cell Biol..

[12] Wu G., Fang Y.-Z., Yang S., Lupton J.R., Turner N.D. (2004). Glutathione metabolism and its implications for health. J. Nutr..

[13] Phan U.T., Arunachalam B., Cresswell P. (2000). Gamma-Interferon-inducibleLysosomal Thiol Reductase (GILT): Maturation, activity, and mechanism of action. J. Biol. Chem..

[14] Kuppusamy P., Li H., Ilangovan G., Cardounel A.J., Zweier J.L., Yamada K., Krishna M.C., Mitchell J.B. (2002). Noninvasive imaging of tumor redox status and its modification by tissue glutathione levels. Cancer Res..

[15] Mollazadeh S., Mackiewicz M., Yazdimamaghani M. (2021). Recent advances in the redox-responsive drug delivery nanoplatforms: A chemical structure and physical property perspective. Mater. Sci. Eng. C.

[16] Du J., Wang L., Han X., Dou J., Jiang X., Yuan J. (2021). Polydopamine/keratin complexes as gatekeepers of mesoporous silica nanoparticles for pH and GSH dual responsive drug delivery. Mater. Lett..

[17] Shen L., Pan S., Niu D., He J., Jia X., Hao J., Gu J., Zhao W., Li P., Li Y. (2019). Facile synthesis of organosilica-capped mesoporous silica nanocarriers with selective redox-triggered drug release properties for safe tumor chemotherapy. Biomater. Sci..

[18] Zhu D., Hu C., Liu Y., Chen F., Zheng Z., Wang X. (2019). Enzyme-/redox-responsive mesoporous silica nanoparticles based on functionalized dopamine as nanocarriers for cancer therapy. ACS Omega.

[19] Chen C., Sun W., Yao W., Wang Y., Ying H., Wang P. (2018). Functional polymeric dialdehyde dextrin network capped mesoporous silica nanoparticles for pH/GSH dual-controlled drug release. RSC Adv..

[20] Luo W., Xu X., Zhou B., He P., Li Y., Liu C. (2019). Formation of enzymatic/redox-switching nanogates on mesoporous silica nanoparticles for anticancer drug delivery. Mater. Sci. Eng. C.

[21] Yan H., Dong J., Huang X., Du X. (2021). Protein-gated upconversion nanoparticle-embedded mesoporous silica nanovehicles via diselenide linkages for drug release tracking in real time and tumor chemotherapy. ACS Appl. Mater. Interfaces.

[22] Li R., Mei X., Li X., Zhang C., Ruan L. (2021). A bolt-like-blocking nanovalve on mesoporous silica nanoparticles for controlled release. Microporous Mesoporous Mater..

[23] Lin J.T., Liu Z.K., Zhu Q.L., Rong X.H., Liang C.L., Wang J., Ma D., Sun J., Wang G.H. (2017). Redox-responsive nanocarriers for drug and gene co-delivery based on chitosan derivatives modified mesoporous silica nanoparticles. Colloids Surfaces B Biointerfaces.

[24] Zhao S., Xu M., Cao C., Yu Q., Zhou Y., Liu J. (2017). A redox-responsive strategy using mesoporous silica nanoparticles for co-delivery of siRNA and doxorubicin. J. Mater. Chem. B.

[25] Li L., Lan S., Ma D. (2020). Ultrastable and versatile layer-by-layer coating based on kinetically trapped host–guest complexation for mesoporous silica nanoparticles. Part. Part. Syst. Charact..

[26] Liu J., Chang B., Li Q., Xu L., Liu X., Wang G., Wang Z., Wang L. (2019). Redox-Responsive dual drug delivery nanosystem suppresses cancer repopulation by abrogating doxorubicin-promoted cancer stemness, metastasis, and drug resistance. Adv. Sci..

[27] Qu H., Yang L., Yu J., Dong T., Rong M., Zhang J., Xing H., Wang L., Pan F., Liu H. (2017). A redox responsive controlled release system using mesoporous silica nanoparticles capped with Au nanoparticles. RSC Adv..

[28] Qiu L., Zhao Y., Li B., Wang Z., Cao L., Sun L. (2017). Triple-stimuli (protease/redox/pH) sensitive porous silica nanocarriers for drug delivery. Sens. Actuators B Chem..

[29] Gao Y., Zhong S., Xu L., He S., Dou Y., Zhao S., Chen P., Cui X. (2019). Mesoporous silica nanoparticles capped with graphene quantum dots as multifunctional drug carriers for photo-thermal and redox-responsive release. Microporous Mesoporous Mater..

[30] Yang Y., Lin Y., Di D., Zhang X., Wang D., Zhao Q., Wang S. (2017). Gold nanoparticle-gated mesoporous silica as redox-triggered drug delivery for chemo-photothermal synergistic therapy. J. Colloid Interface Sci..

[31] Guisasola E., Baeza A., Talelli M., Arcos D., Moros M., De La Fuente J.M., Vallet-Regí M. (2015). Magnetic-responsive release controlled by hot spot effect. Langmuir.

[32] Villaverde G., Gómez-Graña S., Guisasola E., García I., Hanske C., Liz-Marzán L.M., Baeza A., Vallet-Regí M. (2018). Targeted chemo-photothermal therapy: A nanomedicine approximation to selective melanoma treatment. Part. Part. Syst. Charact..

[33] Forman H.J., Zhang H., Rinna A. (2009). Glutathione: Overview of its protective roles, measurement, and biosynthesis. Mol. Asp. Med..

[34] Wu Y., Xu Z., Sun W., Yang Y., Jin H., Qiu L., Chen J., Chen J. (2019). Co-responsive smart cyclodextrin-gated mesoporous silica nanoparticles with ligand-receptor engagement for anti-cancer treatment. Mater. Sci. Eng. C.

[35] Gisbert-Garzarán M., Lozano D., Matsumoto K., Komatsu A., Manzano M., Tamanoi F., Vallet-Regí M. (2021). Designing mesoporous silica nanoparticles to overcome biological barriers by incorporating targeting and endosomal escape. ACS Appl. Mater. Interfaces.

[36] Bhavsar D.B., Patel V., Sawant K.K. (2020). Design and characterization of dual responsive mesoporous silica nanoparticles for breast cancer targeted therapy. Eur. J. Pharm. Sci..

[37] Cui Y., Deng R., Li X., Wang X., Jia Q., Bertrand E., Meguellati K., Yang Y.-W. (2019). Temperature-sensitive polypeptide brushes-coated mesoporous silica nanoparticles for dual-responsive drug release. Chin. Chem. Lett..

[38] Birlik Demirel G., Aygul E., Dag A., Atasoy S., Cimen Z., Cetin B. (2020). Folic acid-conjugated pH and redox-sensitive ellipsoidal hybrid magnetic nanoparticles for dual-triggered drug release. ACS Appl. Bio Mater..

[39] Liu J., Liu X., Yuan Y., Li Q., Chang B., Xu L., Cai B., Qi C., Li C., Jiang X. (2018). Supramolecular modular approach toward conveniently constructing and multifunctioning a pH/redox dual-responsive drug delivery nanoplatform for improved cancer chemotherapy. ACS Appl. Mater. Interfaces.

[40] Zhang L., Wei F., Al-Ammari A., Sun D. (2020). An optimized mesoporous silica nanosphere-based carrier system with chemically removable Au nanoparticle caps for redox-stimulated and targeted drug delivery. Nanotechnology.

[41] Wan L., Chen Z., Deng Y., Liao T., Kuang Y., Liu J., Duan J., Xu Z., Jiang B., Li C. (2020). A novel intratumoral pH/redox-dual-responsive nanoplatform for cancer MR imaging and therapy. J. Colloid Interface Sci..

[42] Chen Z., Wan L., Yuan Y., Kuang Y., Xu X., Liao T., Liu J., Xu Z.Q., Jiang B., Li C. (2020). pH/GSH-dual-sensitive hollow mesoporous silica nanoparticle-based drug delivery system for targeted cancer therapy. ACS Biomater. Sci. Eng..

[43] Chen M., Hu J., Wang L., Li Y., Zhu C., Chen C., Shi M., Ju Z., Cao X., Zhang Z. (2020). Targeted and redox-responsive drug delivery systems based on carbonic anhydrase IX-decorated mesoporous silica nanoparticles for cancer therapy. Sci. Rep..

[44] Zhuang J., Chen S., Hu Y., Yang F., Huo Q., Xie N. (2021). Tumour-targeted and redox-responsive mesoporous silica nanoparticles for controlled release of doxorubicin and an siRNA against metastatic breast cancer. Int. J. Nanomed..

[45] Cheng Y.J., Zhang A.Q., Hu J.J., He F., Zeng X., Zhang X.Z. (2017). Multifunctional peptide-amphiphile end-capped mesoporous silica nanoparticles for tumor targeting drug delivery. ACS Appl. Mater. Interfaces.

[46] Cai Y., Deng T., Pan Y., Zink J.I. (2020). Use of ferritin capped mesoporous silica nanoparticles for redox and pH triggered drug release In Vitro and In Vivo. Adv. Funct. Mater..

[47] Zhou J., Li M., Lim W.Q., Luo Z., Phua S.Z.F., Huo R., Li L., Li K., Dai L., Liu J. (2018). A Transferrin-conjugated hollow nanoplatform for redox-controlled and targeted chemotherapy of tumor with reduced inflammatory reactions. Theranostics.

[48] Chen X., Sun H., Hu J., Han X., Liu H., Hu Y. (2017). Transferrin gated mesoporous silica nanoparticles for redox-responsive and targeted drug delivery. Colloids Surf. B Biointerfaces.

[49] Liu M., Fu M., Yang X., Jia G., Shi X., Ji J., Liu X., Zhai G. (2020). Paclitaxel and quercetin co-loaded functional mesoporous silica nanoparticles overcoming multidrug resistance in breast cancer. Colloids Surf. B Biointerfaces.

[50] Huang L., Liu J., Gao F., Cheng Q., Lu B., Zheng H., Xu H., Xu P., Zhang X., Zeng X. (2018). A dual-responsive, hyaluronic acid targeted drug delivery system based on hollow mesoporous silica nanoparticles for cancer therapy. J. Mater. Chem. B.

[51] Palanikumar L., Kim J., Oh J.Y., Choi H., Park M.H., Kim C., Ryu J.H. (2018). Hyaluronic acid-modified polymeric gatekeepers on biodegradable mesoporous silica nanoparticles for targeted cancer therapy. ACS Biomater. Sci. Eng..

[52] Wang Y., Cui Y., Zhao Y., He B., Shi X., Di D., Zhang Q., Wang S. (2017). Fluorescent carbon dot-gated multifunctional mesoporous silica nanocarriers for redox/enzyme dual-responsive targeted and controlled drug delivery and real-time bioimaging. Eur. J. Pharm. Biopharm..

[53] Zhao Q., Wang S., Yang Y., Li X., Di D., Zhang C., Jiang T., Wang S. (2017). Hyaluronic acid and carbon dots-gated hollow mesoporous silica for redox and enzyme-triggered targeted drug delivery and bioimaging. Mater. Sci. Eng. C.

[54] Narayan R., Nayak U.Y., Raichur A.M., Garg S. (2018). Mesoporous silica nanoparticles: A comprehensive review on synthesis and recent advances. Pharmaceutics.

[55] Rosenholm J.M., Mamaeva V., Sahlgren C., Lindén M. (2012). Nanoparticles in targeted cancer therapy: Mesoporous silica nanoparticles entering preclinical development stage. Nanomedicine.

[56] Croissant J.G., Fatieiev Y., Khashab N.M. (2017). Degradability and clearance of silicon, organosilica, silsesquioxane, silica mixed oxide, and mesoporous silica nanoparticles. Adv. Mater..

[57] Du X., Li X., Xiong L., Zhang X., Kleitz F., Qiao S.Z. (2016). Mesoporous silica nanoparticles with organo-bridged silsesquioxane framework as innovative platforms for bioimaging and therapeutic agent delivery. Biomaterials.

[58] Yang Y., Niu Y., Zhang J., Meka A.K., Zhang H., Xu C., Lin C.X.C., Yu M., Yu C. (2015). Biphasic synthesis of large-pore and well-dispersed benzene bridged mesoporous organosilica nanoparticles for intracellular protein delivery. Small.

[59] Wang K., Li X., Wang H., Lu H., Di D., Zhao Q., Wang S. (2021). Evaluation on redox-triggered degradation of thioether-bridged hybrid mesoporous organosilica nanoparticles. Colloids Surf. A Physicochem. Eng. Asp..

[60] Li X., Chen Y., Zhang X., Zhao Y. (2020). Fabrication of biodegradable auto-fluorescent organosilica nanoparticles with dendritic mesoporous structures for pH/redox-responsive drug release. Mater. Sci. Eng. C.

[61] Hadipour Moghaddam S.P., Saikia J., Yazdimamaghani M., Ghandehari H. (2017). Redox-responsive polysulfide-based biodegradable organosilica nanoparticles for delivery of bioactive agents. ACS Appl. Mater. Interfaces.

[62] Song S., Li X., Ji Y., Lv R., Wu L., Wang H., Cao M., Xu Z. (2021). GSH/pH dual-responsive and HA-targeting nano-carriers for effective drug delivery and controlled release. J. Drug Deliv. Sci. Technol..

[63] Yu L., Chen Y., Lin H., Du W., Chen H., Shi J. (2018). Ultrasmall mesoporous organosilica nanoparticles: Morphology modulations and redox-responsive biodegradability for tumor-specific drug delivery. Biomaterials.

[64] Hadipour Moghaddam S.P., Yazdimamaghani M., Ghandehari H. (2018). Glutathione-sensitive hollow mesoporous silica nanoparticles for controlled drug delivery. J. Control. Release.

[65] Chen Q., Chen Y., Zhang W., Huang Q., Hu M., Peng D., Peng C., Wang L., Chen W. (2020). Acidity and glutathione dual-responsive polydopamine-coated organic-inorganic hybrid hollow mesoporous silica nanoparticles for controlled drug delivery. ChemMedChem.

[66] Zhang Q., Shen C., Zhao N., Xu F.J. (2017). Redox-responsive and drug-embedded silica nanoparticles with unique self-destruction features for efficient gene/drug codelivery. Adv. Funct. Mater..

[67] Mai N.X.D., Birault A., Matsumoto K., Ta H.K.T., Intasa-ard S.G., Morrison K., Thang P.B., Doan T.L.H., Tamanoi F. (2020). Biodegradable periodic mesoporous organosilica (BPMO) loaded with daunorubicin: A promising nanoparticle-based anticancer drug. ChemMedChem.

[68] Tamanoi F., Chinnathambi S., Laird M., Komatsu A., Birault A., Takata T., Doan T.L.H., Mai N.X.D., Raitano A., Morrison K. (2021). Construction of boronophenylalanine-loaded biodegradable periodic mesoporous organosilica nanoparticles for BNCT cancer therapy. Int. J. Mol. Sci..

[69] Vu B.T., Shahin S.A., Croissant J., Fatieiev Y., Matsumoto K., Le-Hoang Doan T., Yik T., Simargi S., Conteras A., Ratliff L. (2018). Chick chorioallantoic membrane assay as an in vivo model to study the effect of nanoparticle-based anticancer drugs in ovarian cancer. Sci. Rep..

[70] Paris J.L., Colilla M., Izquierdo-barba I., Manzano M., Vallet-Regí M. (2017). Tuning mesoporous silica dissolution in physiological environments: A review. J. Mater. Sci..

[71] Croissant J., Cattoën X., Man M.W.C., Gallud A., Raehm L., Trens P., Maynadier M., Durand J.O. (2014). Biodegradable ethylene-bis(propyl)disulfide-based periodic mesoporous organosilica nanorods and nanospheres for efficient In-Vitro drug delivery. Adv. Mater..

[72] Li X., Zhang X., Zhao Y., Sun L. (2020). Fabrication of biodegradable Mn-doped mesoporous silica nanoparticles for pH/redox dual response drug delivery. J. Inorg. Biochem..

[73] Shao D., Li M., Wang Z., Zheng X., Lao Y.H., Chang Z., Zhang F., Lu M., Yue J., Hu H. (2018). Bioinspired diselenide-bridged mesoporous silica nanoparticles for dual-responsive protein delivery. Adv. Mater..

